# Supplementary material for: Cancer cells copy migratory behavior and exchange signaling networks via extracellular vesicles
Source: EMBO J. 2018 Jun 15;37(15):e98357. doi: 10.15252/embj.201798357 (PMC6068466; doi:10.15252/embj.201798357)
Supplement: Supplementary file 1 — Appendix [file EMBJ-37-e98357-s001.pdf]

Contents

Appendix Figure S1 Legend **2**

Appendix Figure S1 **3**

Appendix Figure S2 Legend **4**

Appendix Figure S2 **5**

Appendix Figure S1. Estimation of percentage of cancer cells within B16F1 and B16F10 tumors. (a) Representative images of sections of B16F1 (top) and B16F10 (bottom) reporter^{+} tumors for DsRed and DAPI staining. Nuclear regions were assessed for expression of DsRed and all nuclei were scored positive (cancer cell nucleus, yellow edge) or scored negative (stromal cell nucleus, white edge and marked with an asterisk) for DsRed. Scale bars are 50µm. (b) Quantification of 4-5 tumor regions per tumor for 3 mice per model, colors indicate tumor regions per mouse.

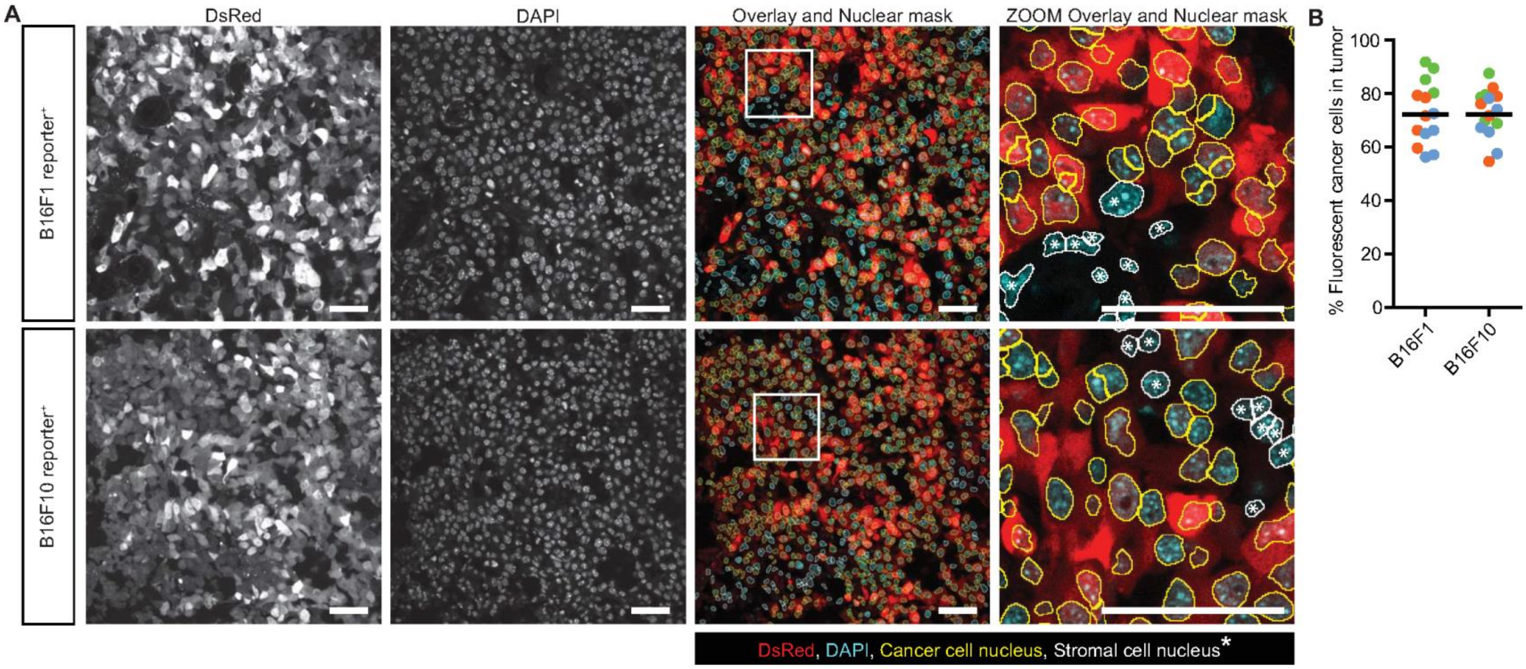
A
DsRed
DAPI
Overlay and Nuclear mask
ZOOM Overlay and Nuclear mask
B16F1 reporter+
B16F10 reporter+
DsRed, DAPI, Cancer cell nucleus, Stromal cell nucleus*
B
% Fluorescent cancer cells in tumor
0
20
40
60
80
100
B16F1
B16F10

Appendix Figure S2. Predicted molecular weight of detected proteins per gel band. For each gel block per sample, the frequency of the predicted molecular weight of all identified proteins was plotted. Average molecular weight (in kDa, +/- SD) per fraction is displayed above each graph.

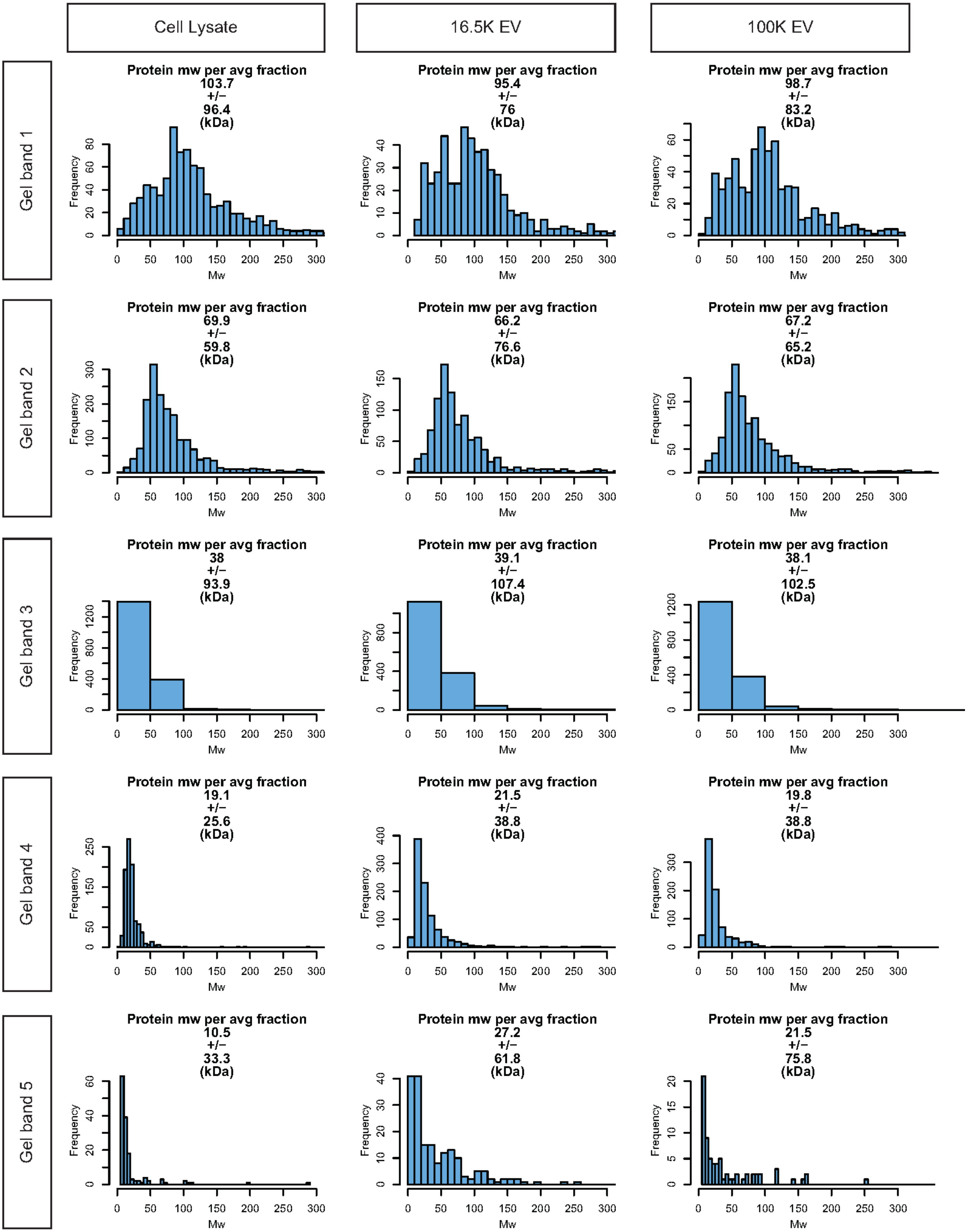
Cell Lysate
16.5K EV
100K EV
Gel band 1
Gel band 2
Gel band 3
Gel band 4
Gel band 5
Protein mw per avg fraction 103.7 +/− 96.4 (kDa)
Protein mw per avg fraction 95.4 +/− 76 (kDa)
Protein mw per avg fraction 98.7 +/− 83.2 (kDa)
Protein mw per avg fraction 69.9 +/− 59.8 (kDa)
Protein mw per avg fraction 66.2 +/− 76.6 (kDa)
Protein mw per avg fraction 67.2 +/− 65.2 (kDa)
Protein mw per avg fraction 38 +/− 93.9 (kDa)
Protein mw per avg fraction 39.1 +/− 107.4 (kDa)
Protein mw per avg fraction 38.1 +/− 102.5 (kDa)
Protein mw per avg fraction 19.1 +/− 25.6 (kDa)
Protein mw per avg fraction 21.5 +/− 38.8 (kDa)
Protein mw per avg fraction 19.8 +/− 38.8 (kDa)
Protein mw per avg fraction 10.5 +/− 33.3 (kDa)
Protein mw per avg fraction 27.2 +/− 61.8 (kDa)
Protein mw per avg fraction 21.5 +/− 75.8 (kDa)
Frequency
Mw
