# Supplementary material for: Cancer cells copy migratory behavior and exchange signaling networks via extracellular vesicles
Source: EMBO J. 2018 Jun 15;37(15):e98357. doi: 10.15252/embj.201798357 (PMC6068466; doi:10.15252/embj.201798357)
Supplement: Supplementary file 2 — Expanded View Figures PDF [file EMBJ-37-e98357-s002.pdf]

Expanded View Figures

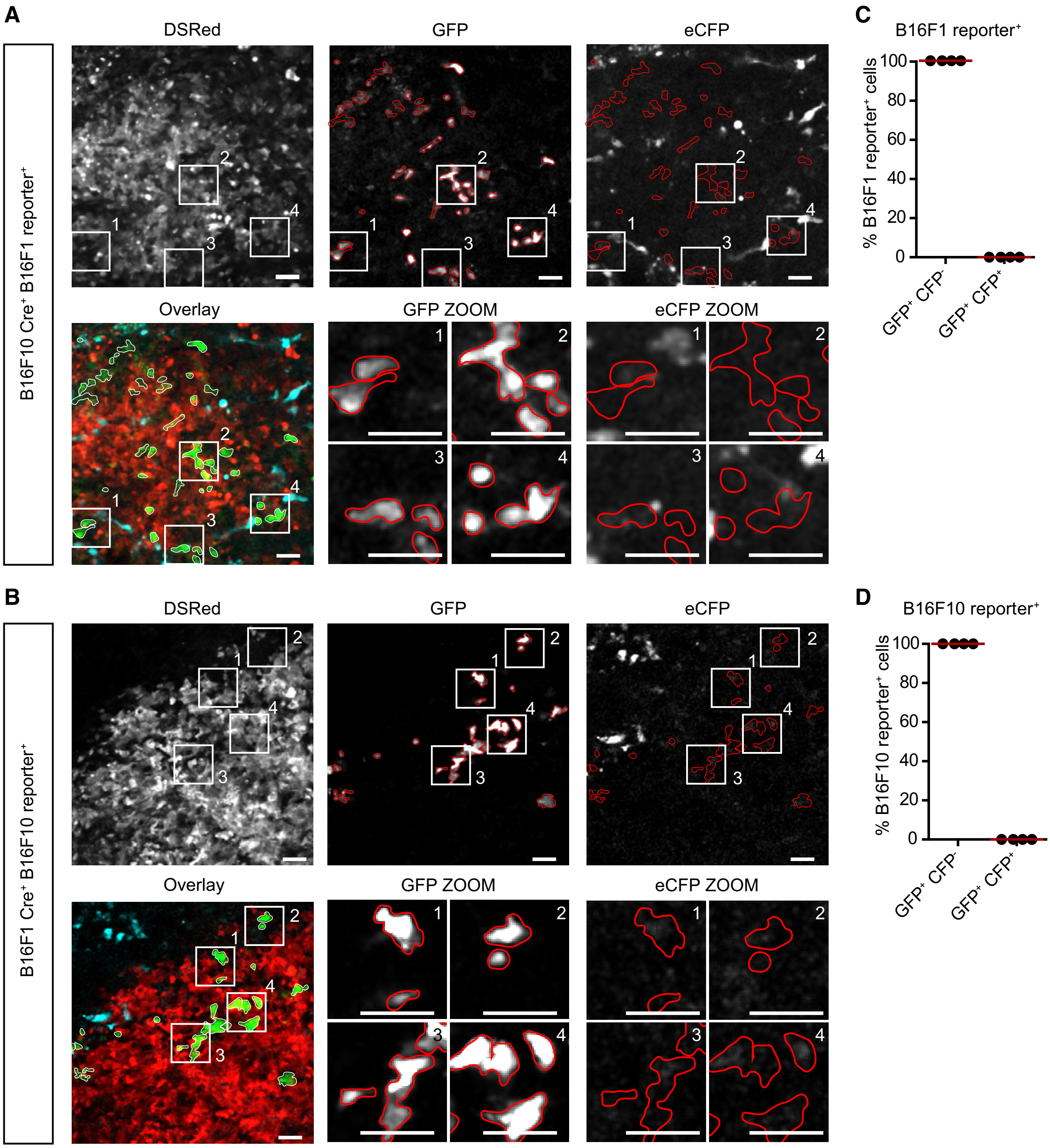

Figure EV1. GFP^{+} reporter cells are negative for expression of CFP.

A, B Representative images of eGFP^{+} reporter cells from B16F10 Cre^{+} B16F1 reporter^{+} tumor mixes and B16F1 Cre^{+} B16F10 reporter^{+} tumor mixes. Displayed are the overlay and single channels for DsRed, GFP, and CFP, and red outlines indicate borders of GFP-expressing cells. Numbered zooms for four regions of interest are displayed for the GFP and CFP channels below the overview image. Scale bars are 50 µm.

C, D A total of 638 eGFP cells were assessed for expression of CFP; B16F1 reporter^{+} GFP^{+} cells; n = 331 cells in four mice total, B16F10 reporter^{+} GFP^{+} cells, n = 307 cells in four mice total. No co-expression of GFP and CFP was observed.

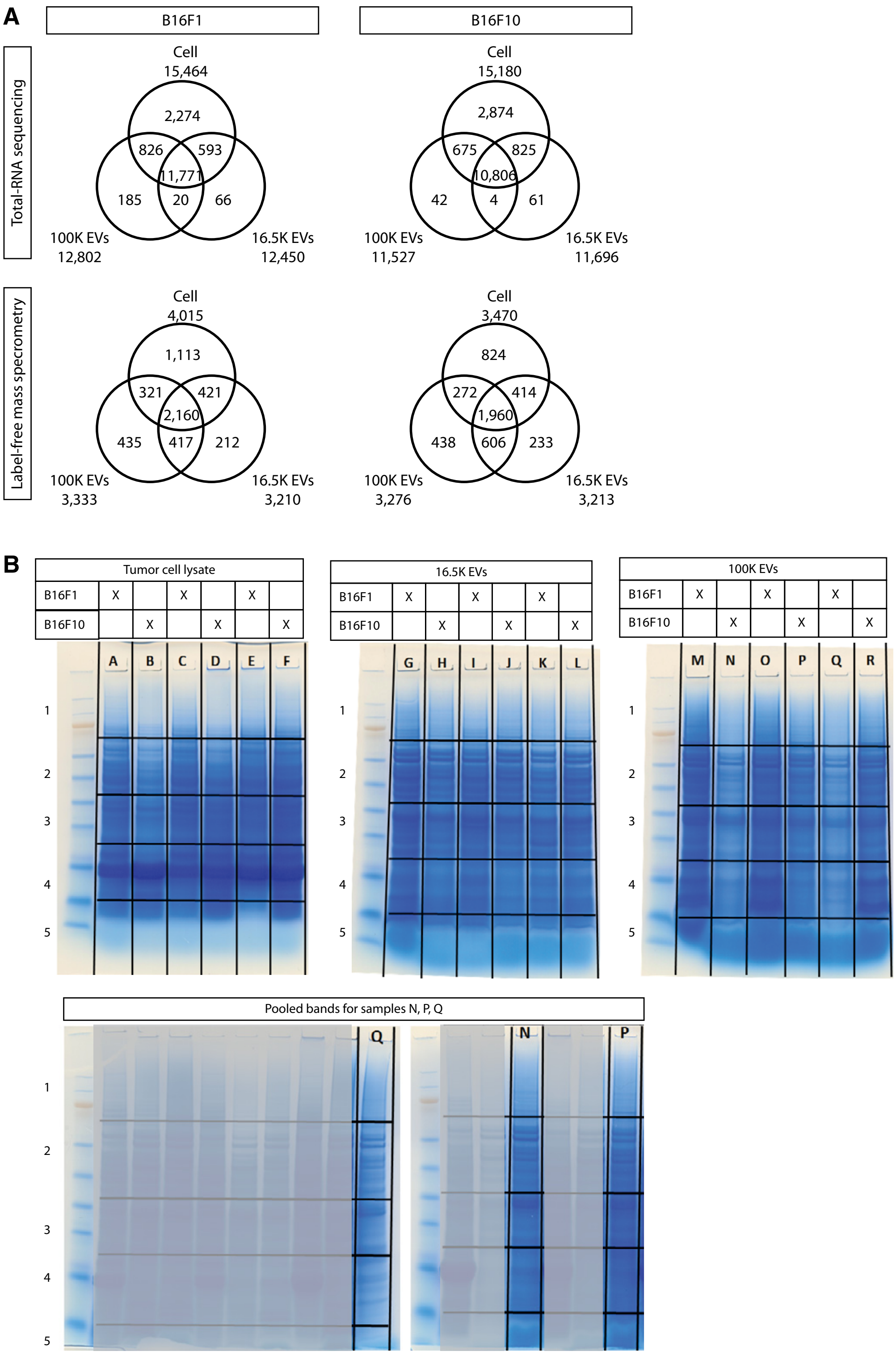

Figure EV2. Detection of EV-associated RNAs and proteins from B16F1- and B16F10-derived EVs.

A Venn diagrams displaying the number of transcripts (top) and proteins (bottom) identified in all three replicates of the cells, 16.5K EVs, and 100K EVs in B16F1 (left) and B16F10 (right) tumors.

B Coomassie-stained gels of samples prepped for label-free mass spectrometry, including cutting scheme overlay of individual slices used for protein extraction using in-gel digestion. Samples N, P, and Q were pooled to normalize protein amount per fraction.

© 2018 The Authors

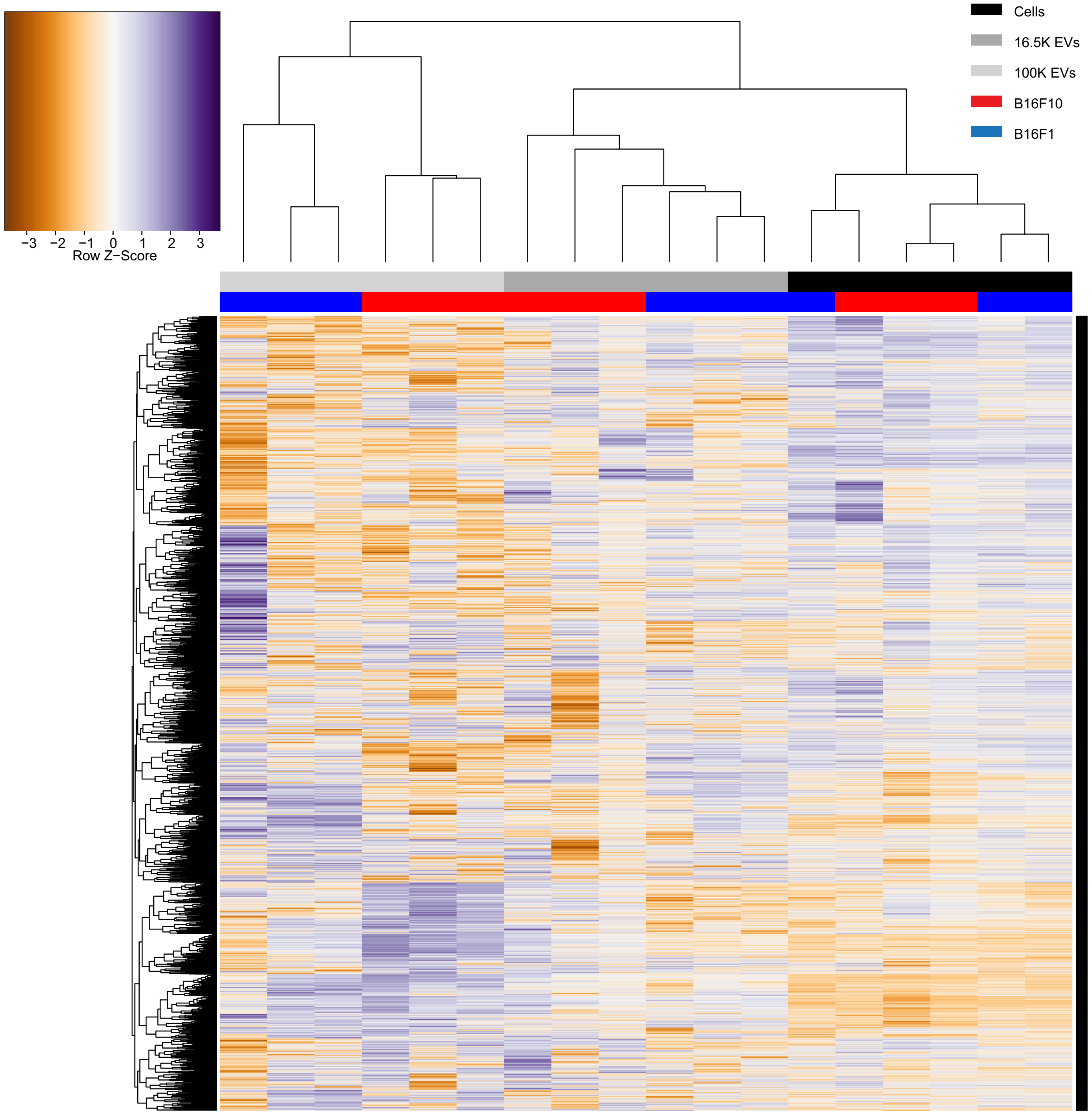

Figure EV3. Unsupervised clustering of cell and EVs samples of the B16F1 and B16F10 model on total RNA expression.

Clustering of RNA-seq data confirms that sample types (i.e., cells, 16.5K EVs, and 100K EVs) are more similar than overall differences between B16F1 and B16F10.

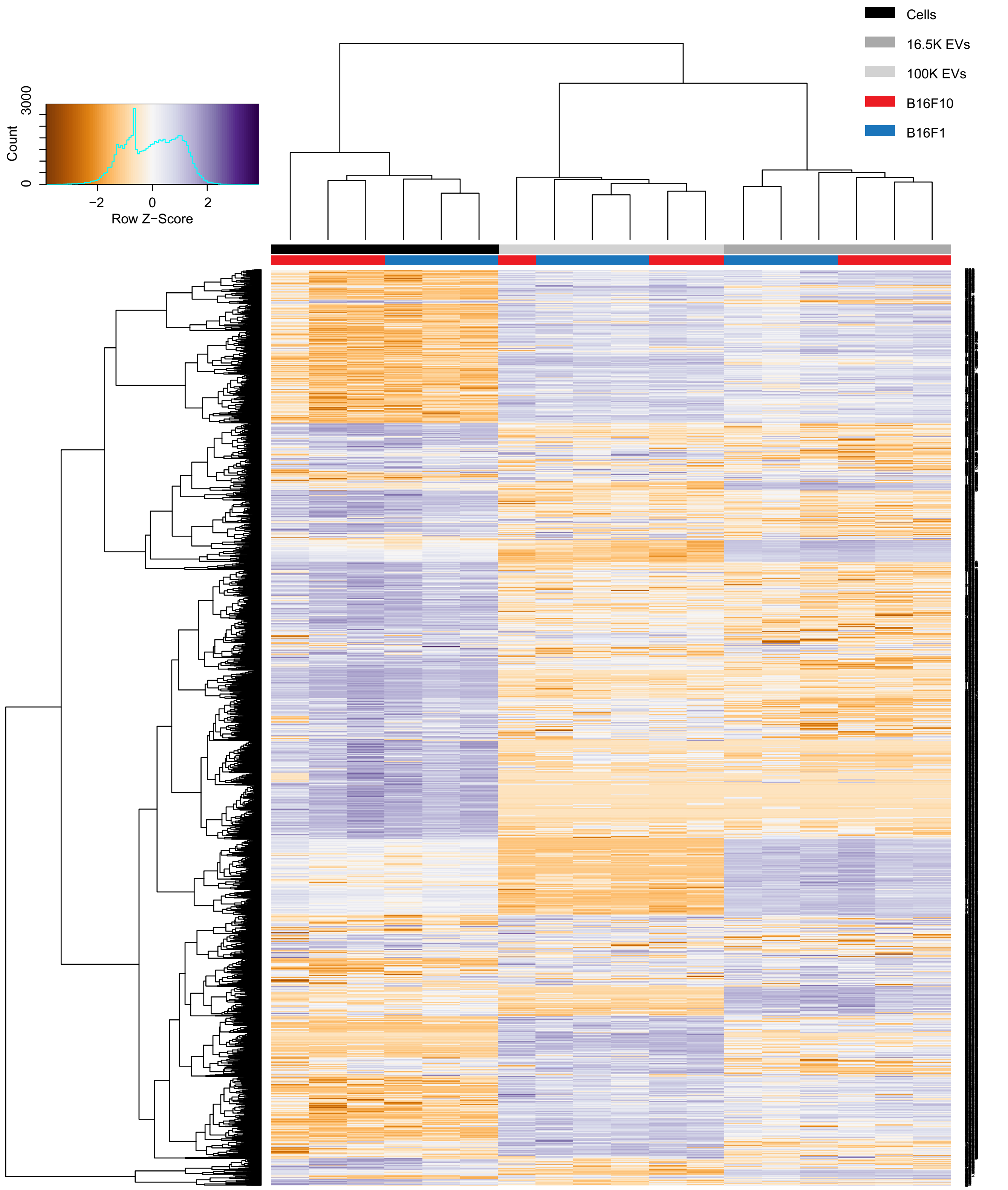

Figure EV4. Unsupervised clustering of cell and EVs samples of the B16F1 and B16F10 model on protein profile.

Clustering of all identified proteins from label-free mass spectrometry confirms that sample types (i.e., cells, 16.5K EVs, and 100K EVs) are more similar than overall differences between B16F1 and B16F10.

 © 2018 The Authors

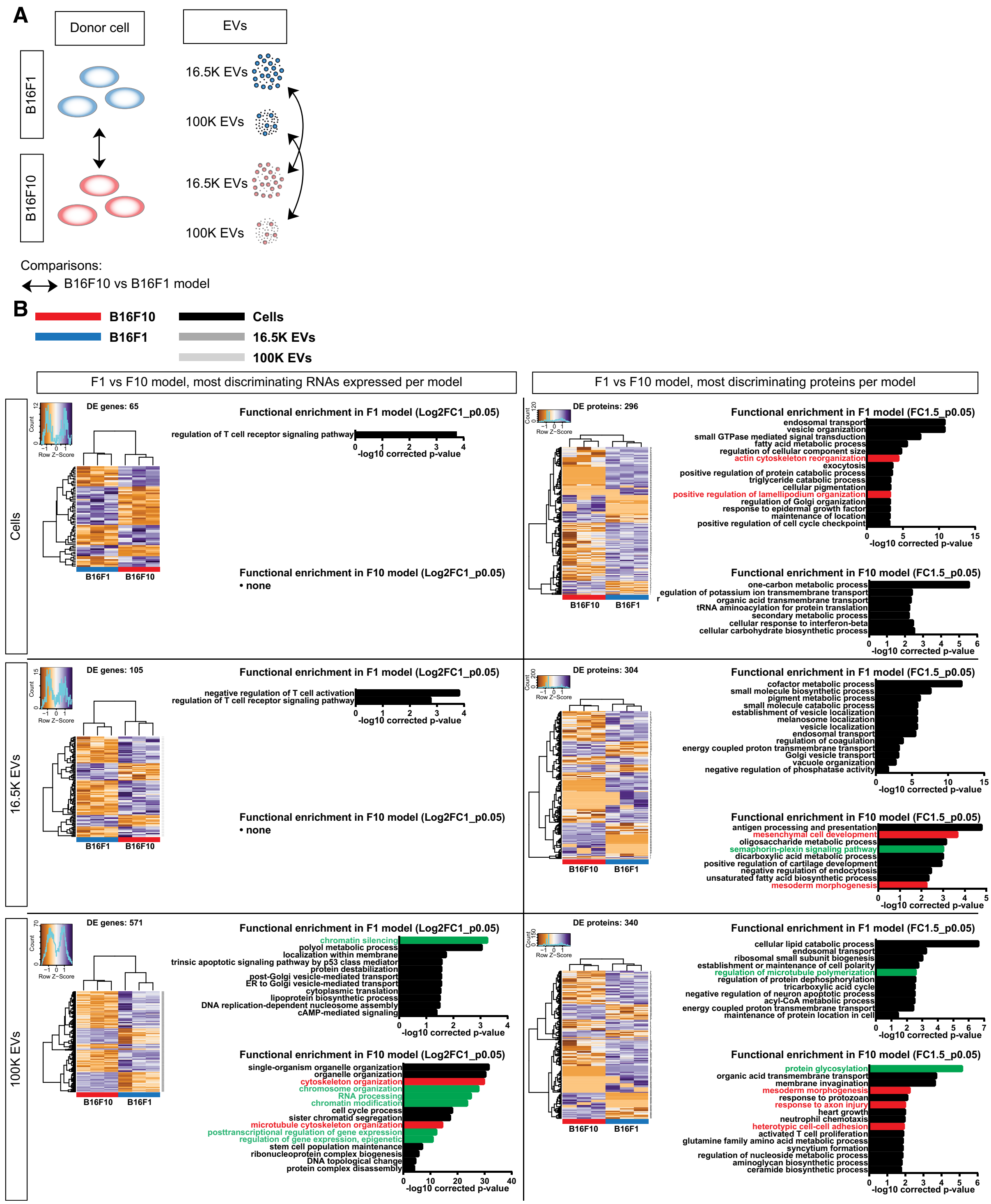

Figure EV5. Differential protein and gene expression between the B16F1 and B16F10 model.

A Cartoon of intra-sample type comparison of protein and RNA profiles.

B Differential expression between cells (top), 16.5K EVs (middle), and 100K EVs (bottom) between the B16F1 and B16F10 model for most discriminating gene expression levels (Log2FC ≥ 1 and $P \leq 0.05$, left) and proteins (FC ≥ 20 and $P \leq 0.01$, right). For every comparison, number of DE genes and proteins, supervised clustering of samples, and gene ontology for more abundant in B16F1 and more in abundant B16F10 are depicted. GO terms related to cell migration are highlighted in red, and indirect influencers of cell migration are highlighted in green. For the cells and 16.5K EV comparison, no functional GO term enrichment is present in the B16F10-specific transcript set.

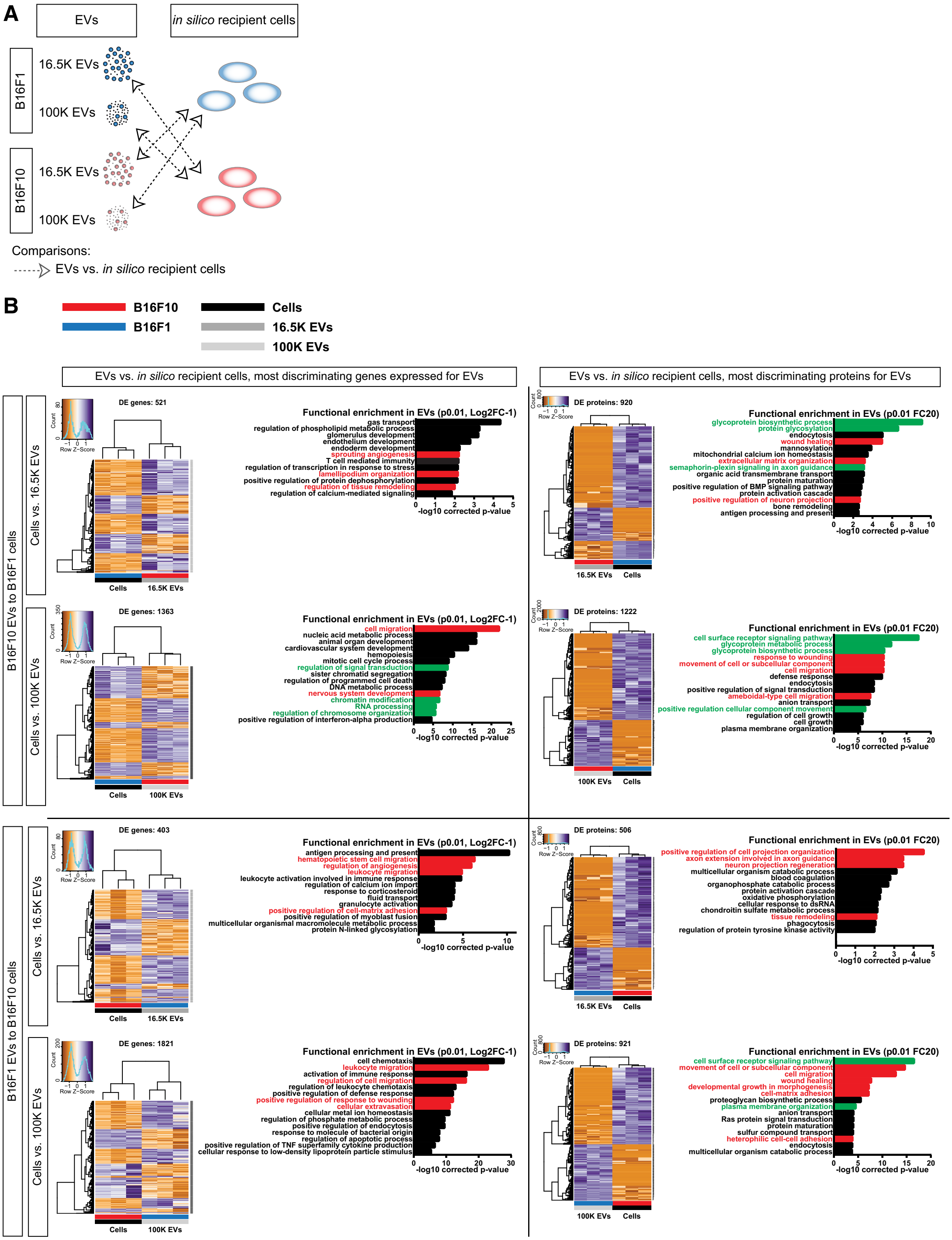

Figure EV6. Differential protein and gene expression between EVs and recipient cells across models.

A Cartoon of the comparison of EVs to cells across models.

B Differential expression between B16F10 EVs and B16F1 cells (top) and B16F1 EVs and B16F10 cells (bottom) for most discriminating gene expression levels (Log2FC ≥ 1 and $P \leq 0.05$, left) and proteins (FC ≥ 20 and $P \leq 0.01$, right). For every comparison, number of DE proteins, supervised clustering of samples, and gene ontology for enrichment in EVs are depicted. GO terms related to cell migration are highlighted in red, and indirect influencers of cell migration are highlighted in green.

 © 2018 The Authors
